# Highly sensitive reporter cell line for detection of interferon types I–III and their neutralization by antibodies

**DOI:** 10.1002/eji.202451325

**Published:** 2024-10-04

**Authors:** Kevin Groen, Roger Kuratli, Lauren Sar, Andri Vasou, Michael Huber, David J. Hughes, Benjamin G. Hale

**Affiliations:** ^1^ Institute of Medical Virology University of Zurich Zurich Switzerland; ^2^ Biomedical Sciences Research Complex University of St. Andrews St. Andrews United Kingdom

**Keywords:** Autoantibodies, Innate immunity, Interferon, Luciferase, Reporter cells

## Abstract

Interferons (IFNs) are a critical component of innate immune defenses and limit viral disease severity. To advance studies on IFNs and their neutralization by pathogenic autoantibodies, we generated a Renilla luciferase‐based reporter cell line capable of detecting the activities of IFN‐Is, IFN‐II, and IFN‐IIIs. The reporter cell line exhibits a 125‐ to 2000‐fold higher sensitivity to IFNs than a commonly used alternative biological reporter system and allows for a rapid and simple live‐cell workflow for detecting low titer amounts of neutralizing anti‐IFN antibodies.

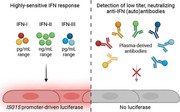

The human IFN system constitutes a critical component of innate immunity. Upon detection of infection, cells initiate the production and secretion of IFN cytokines (IFN‐I, IFN‐II, and IFN‐III types) that trigger the expression of IFN‐stimulated genes (ISGs), causing cells to enter an antiviral state. IFN‐Is (e.g. IFN‐α, IFN‐β, and IFN‐ω) and IFN‐IIIs (e.g. IFN‐λ1‐4) are most important to first‐line antiviral defenses and signal via type‐specific receptors expressed on cell surfaces [1]. IFN system deficiencies can seriously exacerbate the severity of infections [2], and a functional IFN deficiency caused by autoantibodies (autoAbs) neutralizing IFN‐Is promotes replication and disease caused by several viral pathogens [3, 4, 5, 6, 7, 8]. In addition, IFN‐II (IFN‐γ) autoAbs have been described to promote susceptibility to some mycobacterial, fungal, and Gram‐negative bacterial infections, and can increase the likelihood of herpes zoster [9]. While IFN‐III autoAbs have also been identified, they have yet to be linked to a specific disease phenotype [10]. Identification of individuals harboring neutralizing anti‐IFN autoAbs is critical to understand susceptibility to severe infections and requires highly sensitive and robust assays.

Previously, the neutralizing capacity of anti‐IFN‐I autoAbs has been detected by transfecting 293T cells with plasmids expressing Firefly luciferase under the control of an ISG (*MX1*) promoter, followed by stimulation with recombinant IFNs preincubated with patient plasmas. Neutralization is then inferred from reduced levels of IFN‐induced luciferase activity 16–24 h later [6, 11]. However, this system has several caveats: (1) transfection adds 24–48 h to assay duration and increases variability (controlled by co‐transfecting a constitutively‐expressed reporter); (2) relatively high IFN amounts are required, limiting assay sensitivity; (3) peak IFN‐induced luciferase requires 24 h, extending assay duration; and (4) 293T cells are nonresponsive to IFN‐IIIs, prohibiting their detection of anti‐IFN‐III autoAbs. We therefore developed highly sensitive reporter cells for the detection of IFN‐Is, IFN‐II, and IFN‐IIIs based on A549s, which respond to all IFN types [12]. A549 cells were modified to harbor a gene cassette for monocistronic expression of Renilla luciferase and puromycin resistance downstream of the human *ISG15* promoter (prISG15) (Fig. 1A)[13]. The *ISG15* promoter was chosen for its inducibility by all IFNs, with *ISG15* levels being maintained for at least 48 h [14]. Renilla luciferase was chosen as the reporter enzyme due to the availability of a live‐cell substrate, allowing temporal monitoring of luciferase activity. To enhance sensitivity, Renilla luciferase was expressed with a C‐terminal PEST (peptide rich in proline, glutamate, serine, and threonine) sequence that promotes protein degradation, reducing background luciferase from leaky transcription [15]. Single‐cell clones were stimulated with 1000 international units (IU)/mL of IFN‐α2 or IFN‐λ1, and a clone with high responsiveness was selected for use (Fig. 1B), which was named the A549 IFN‐reporter (AIR) cell line.

**Figure 1:**
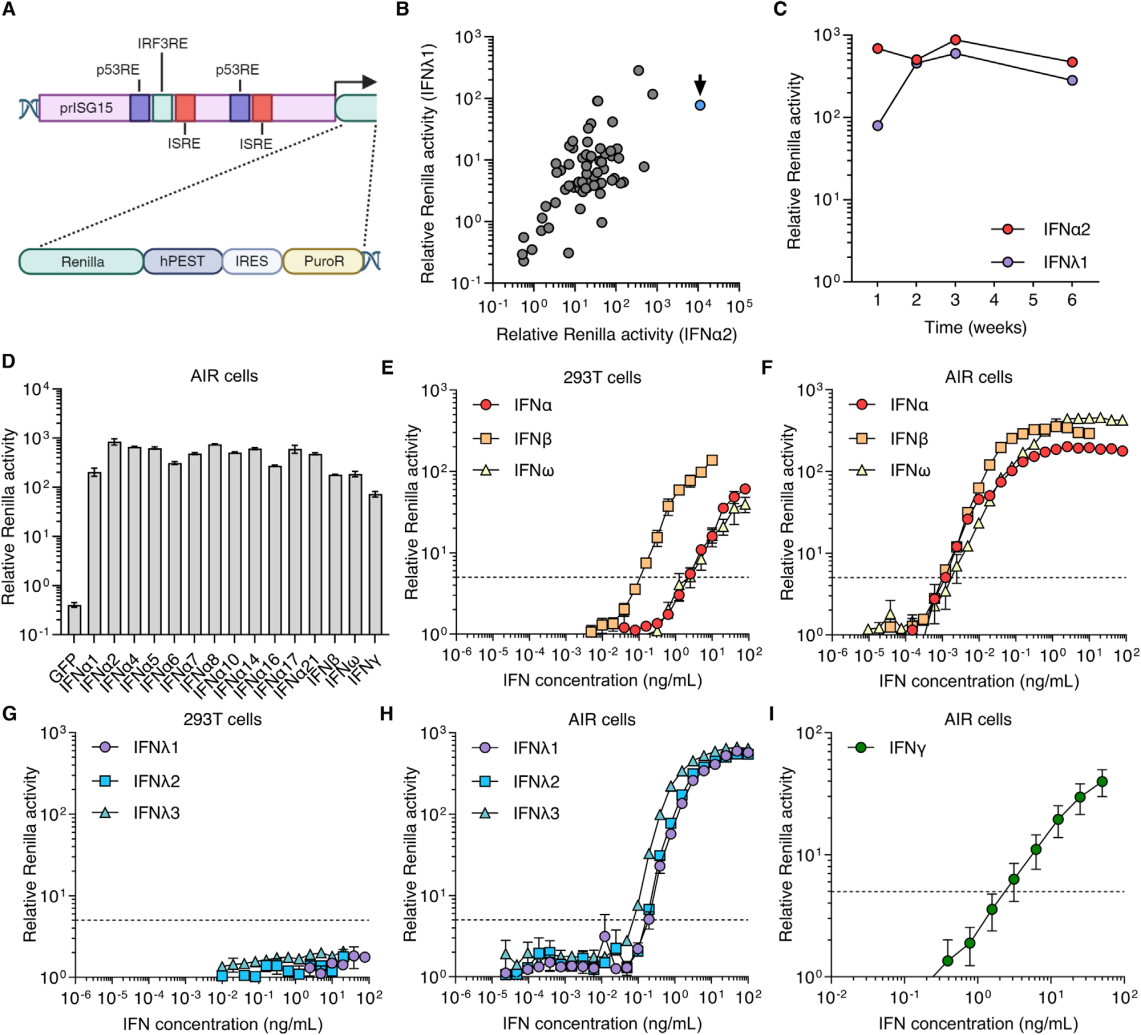
Generation of a highly sensitive A549‐IFN‐reporter (AIR) cell line. (A) Schematic of the gene cassette used to transduce A549 cells, adapted from [13]. Abbreviations: p53RE, p53‐responsive element; IRF3RE, IRF3‐responsive element; ISRE, IFN‐stimulated response element; PuroR, puromycin resistance gene; IRES, internal ribosome entry site; hPEST, peptide‐rich in proline, glutamate, serine, and threonine. (B) Renilla luciferase activity following stimulation of transduced cell clones with IFN‐α2 or IFN‐λ1 (1000 IU/mL) for 24 h. The arrow indicates the clone selected for characterization. (C) Stability of IFN‐α2 and IFN‐λ1 responsiveness (1000 IU/mL, 24 h) in AIR cells after passaging over 6 weeks. (D) Relative Renilla activities of AIR cells stimulated with normalized supernatants from 293T cells transfected with plasmids encoding the indicated HiBiT‐tagged IFN subtypes or GFP control. (E–H) Sensitivity of a standard 293T‐based assay (E, G) and AIR cell assay (F, H) to stimulation with the indicated concentrations of IFN‐Is (α2, β, ω; 24 h; E, F) or IFN‐IIIs (λ1‐3; 24 h; G–H). (I) Sensitivity of AIR cells to stimulation with the indicated IFN‐γ concentrations (24 h). Dashed lines in E–I indicate fivefold luciferase induction compared to non‐stimulated cells. Mean values from *n* = 3 replicates are shown in D–I and error bars represent standard deviations. In all panels, data are representative of at least *n* = 2 similar experiments.

First, we confirmed stable responsiveness of AIR cells to IFN‐α2 or IFN‐λ1 following passaging over 6 weeks (Fig. 1C). In addition, responsiveness to all 12 IFN‐α subtypes, as well as IFN‐β, IFN‐ω, and IFN‐γ, was confirmed (Fig. 1D). We then compared the IFN‐sensitivities of AIR cell assays with those of the standard 293T transfection‐based system. Both reporter systems were stimulated with a concentration range of IFN‐Is or IFN‐IIIs, and IFN‐induced luciferase activities were determined 24 h later. Sensitivity was defined as the lowest IFN dose that induced activity >fivefold above the nonstimulated condition. The 293T system detected 2.5 ng/mL IFN‐α2, 150 pg/mL IFN‐β, and 2.5 ng/mL IFN‐ω (Fig. 1E). By comparison, the AIR system detected as low as 1.2 pg/mL IFN‐α2 or IFN‐β, and 2.4 pg/mL for IFN‐ω (Fig. 1F). While 293T cells did not respond to stimulation with IFN‐IIIs (Fig. 1G), AIR cells responded to 195 pg/mL IFN‐λ1 or IFN‐λ2, and 97.5 pg/mL IFN‐λ3 (Fig. 1H). Additionally, AIR cells responded to IFN‐γ stimulation, although with a lower sensitivity (3.1 ng/mL) (Fig. 1I), which may be a limitation of these cells. Thus, AIR cells display a 125‐ to 2000‐fold higher sensitivity to IFNs than 293Ts, and universally react to all three IFN types.

Next, we compared IFN‐induced luciferase kinetics between the AIR cell system and the 293T‐based system. Using a single dose of each IFN (10 ng/mL IFN‐α or IFN‐ω, 1.21 ng/mL IFN‐β, 50 ng/mL IFN‐λ1‐3), the earliest robust luciferase induction (>fivefold over nonstimulated) in 293Ts occurred 6–8 h poststimulation, and peaked at 24–30 h (Fig. 2A). In contrast, the earliest luciferase signal in AIR cells was detectable after 1 h of stimulation, and peaked after 8 h (Fig. 2B). Importantly, this signal remained stable for 30 h, providing a rapidly‐induced, long‐lasting experimental window compared to the lengthy transfection steps, slow reactivity, and cell lysis requirements in transfection‐based 293T assays.

**Figure 2:**
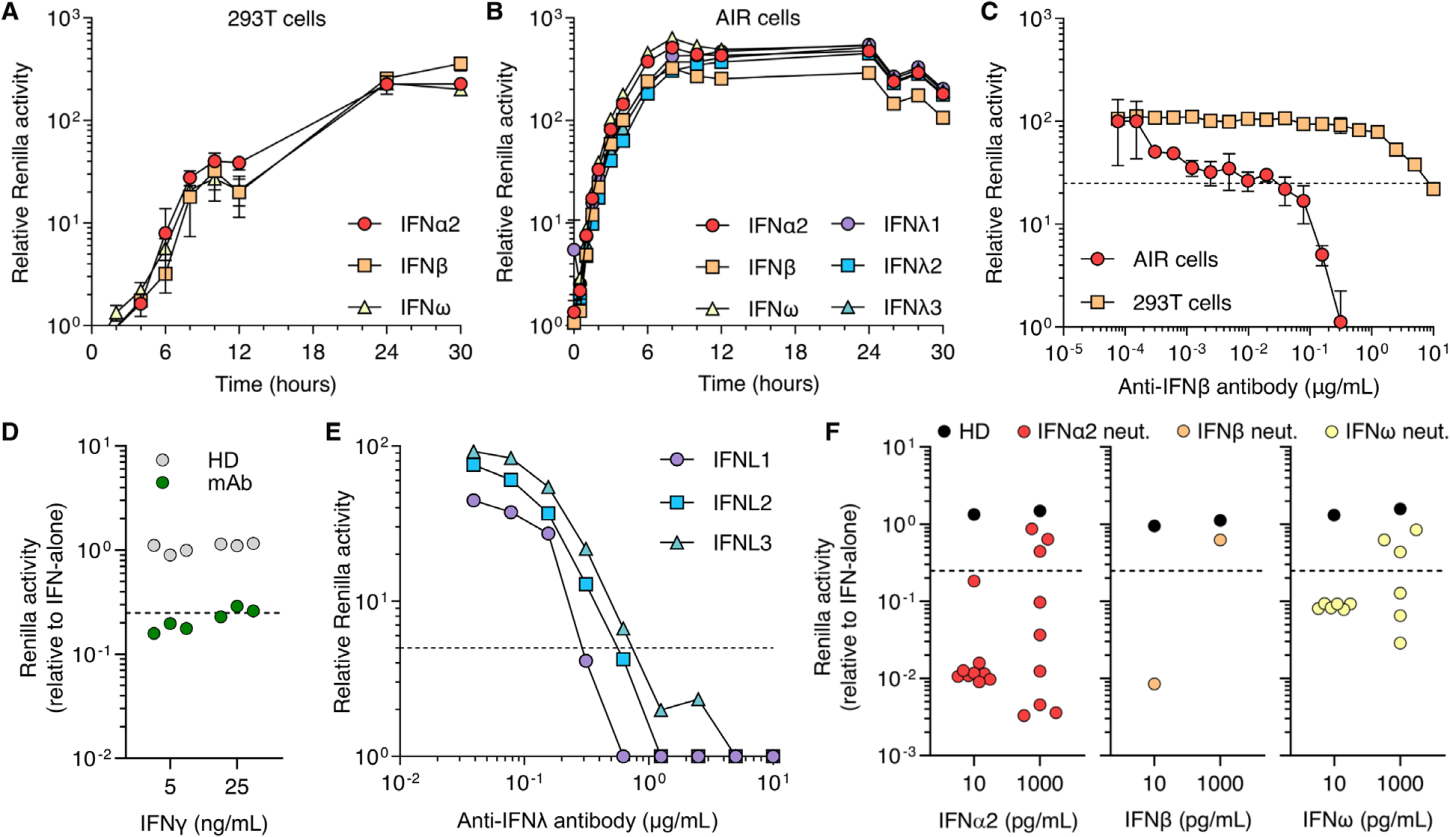
Characterization of AIR cells and their application to detect neutralizing anti‐IFN antibodies. (A, B) Luciferase induction kinetics following stimulation of 293T cells (A) or AIR cells (B) with IFN‐Is (IFN‐α2 and IFN‐ω: 10 ng/mL, IFN‐β: 1.21 ng/mL) or IFN‐IIIs (IFN‐λ1‐3; 50 ng/mL). (C) Sensitivity analysis of 293T cells and AIR cells to detect neutralization of IFN‐β by an anti‐IFN‐β antibody. The indicated concentration of antibody was incubated with 200 pg/mL IFN‐β or 3.6 pg/mL IFN‐β prior to stimulation of 293T or AIR cells, respectively. (D) Use of AIR cells to detect neutralization of IFN‐γ by a 1:100 diluted monoclonal anti‐IFN‐γ antibody. HD, healthy donor plasmas; mAb, monoclonal antibody. (E) Sensitivity analysis of AIR cells to detect neutralization of IFN‐IIIs by their respective antibodies. Indicated concentrations of antibodies were incubated with 10 ng/mL IFN‐λ1, IFN‐λ2, or IFN‐λ3 prior to stimulation of AIR cells. (F) Use of AIR cells to determine the neutralization of IFN‐α2, IFN‐β, or IFN‐ω at the indicated concentrations by previously characterized human patient plasmas (colored dots, plasmas described to neutralize low doses of IFN‐I; black dots, nonneutralizing control plasmas). The dashed lines in C–F indicate the 75% neutralization threshold. For A–C, mean values from *n* = 3 replicates are shown, and error bars represent standard deviations. In all panels, data are representative of at least *n* = 2 similar experiments.

Finally, we assessed the sensitivity of AIR cell assays to detect neutralizing anti‐IFN antibodies. We initially used the lowest amount of IFN‐I which resulted in a 10‐fold induction of luciferase signal over nonstimulated cells (200 pg/mL IFN‐β for 293T cells, 3.6 pg/mL IFN‐β for AIR cells). These IFNβ amounts were incubated with a dilution series of a neutralizing anti‐IFN‐β antibody for 1 h prior to stimulation of each cell system. With a neutralization threshold of 75% reduction, the 293T assay detected 10 µg/mL neutralizing antibody, while AIR cell assays had a 250‐fold improved sensitivity, detecting neutralization with only 39 ng/mL antibody (Fig. 2C). Similar assays demonstrated that AIR cells can be used to detect neutralizing anti‐IFN‐II and IFN‐III antibodies (Fig. 2D and E). Lastly, we selected previously characterized human plasmas shown to neutralize low doses of IFN‐α (200 pg/mL), IFN‐β (40 pg/mL), or IFN‐ω (200 pg/mL) using the 293T assay [11] and demonstrated the improved ability of AIR cells to detect the neutralizing activity of these patient samples against IFN‐I concentrations as low as 10 pg/mL (Fig. 2F).

In summary, the high sensitivity of AIR cells to respond to all IFN types permits their application to detect low titer neutralizing anti‐IFN autoAbs.

## Conflict of interest

The authors declare no financial or commercial conflict of interest.

### Peer review

The peer review history for this article is available at https://publons.com/publon/10.1002/eji.202451325.

**Abbreviations**
AIR: A549 interferon‐reporter
AutoAbs: autoantibodies
ISG: interferon‐stimulated gene
IU: international units
prISG15: promoter of interferon‐stimulated gene 15

## Supporting information



Supporting Information

## Data Availability

The data that support the findings of this study are available from the corresponding author upon reasonable request. Detailed methods can be found in the Supporting Information file.
