# Supplementary material for: Highly sensitive reporter cell line for detection of interferon types I–III and their neutralization by antibodies
Source: Eur J Immunol. 2024 Oct 4;54(12):2451325. doi: 10.1002/eji.202451325 (PMC11628890; doi:10.1002/eji.202451325)
Supplement: Supplementary file 1 — Supporting Information [file EJI-54-2451325-s001.pdf]

Supporting information for Groen *et al*.

Material and methods.

Cells and media. 293T and A549 cells were originally from ATCC, and were cultured in Dulbecco's Modified Eagle's Medium (DMEM; Thermo Fisher Scientific) supplemented with 10% fetal bovine serum (FBS) and 100 U/mL penicillin and streptomycin (Gibco Life Technologies; 15140-122). Cells were grown at 37°C with 5% CO_2.

Human plasma samples and ethics. Anonymized human plasma samples previously categorized as positive or negative for anti-IFN-I autoAbs [1] were used to validate assays. Most samples were originally derived from specimens stored in the Zurich center biobank of the Swiss HIV Cohort study (SHCS, [2]) which covers their use in the current work. Detailed information on the study is available at http://www.shcs.ch. The SHCS has been approved by the ethics committees of all participating institutions (Kantonale Ethikkommission Bern, Ethikkommission des Kantons St. Gallen, Comite Departemental d'Ethique des Specialites Medicales et de Medicine Communataire et de Premier Recours, Kantonale Ethikkommission Zürich, Repubblica et Cantone Ticino–Comitato Ethico Cantonale, Commission Cantonale d'Étique de la Recherche sur l'Être Humain, Ethikkommission beider Basel), and written informed consent has been obtained from all participants. As described previously [3,4], leftover healthy donor plasma samples were also derived from specimens provided by the Zurich Blood Transfusion Service of the Swiss Red Cross and were used with approval of the responsible local ethics committee (Kantonale Ethikkommission Zurich BASEC ID 2021-00437 and 2021-01138). All data were analyzed anonymously.

Cloning of plasmids. Plasmid prISG15-eGFP-IRES-Puro has been described previously [5]. For cloning of plasmid prISG15-Renilla-hPEST-IRES-Puro, the Renilla luciferase sequence was PCR amplified from plasmid pRL-TK-Renilla (Promega, E2241) and the hPEST sequence was PCR amplified from plasmid pcDNA3.1(+)GUG-nLuc-3XFLAG-CL1/PEST (Addgene plasmid #127317; a gift from Jeremy Wilusz [6]). The forward hPEST primer contained a 20 nucleotide tail complementary to the reverse primer used to amplify Renilla luciferase. The two PCR products, with a 20 nucleotide sequence overlap, were fused by PCR using the Renilla forward primer and the hPEST reverse primer, resulting in a Renilla-hPEST PCR product. The forward Renilla primer contained a tail with the XhoI restriction enzyme sequence and the hPEST reverse primer contained the NotI restriction enzyme sequence. This resulted in a final Renilla-hPEST PCR product flanked by XhoI and NotI restriction sequences that were used to replace eGFP in the original vector by standard cloning practices. Plasmids (pcDNA3.1-based) encoding individual full-length secreted IFN subtypes with C-terminal HiBiT tags (Promega), or GFP control, were generated using GeneArt gene synthesis services (Thermo Fisher Scientific).

Lentivirus production, transduction of A549 cells, and selection. 293T cells were seeded in 6-well plates and transfected with the prISG15-Renilla-hPEST-IRES-Puro construct together with psPAX2 and pMD2.G (Addgene plasmids #12259 and #12260; gifts from Didier Trono) at a ratio of 2:1:1. FuGene HD (Promega; E2311) was used as the transfection reagent according to the manufacturer's instructions. At 48h post-transfection, supernatants were harvested and filtered through a 0.45 µm syringe filter. Sub-confluent A549 cells were subsequently transduced with 2 mL of the filtered lentivirus-containing supernatant supplemented with 8 µg/mL polybrene (Sigma-Aldrich; 107689) for 24h. Transduced cells were then stimulated with 1000 IU/mL IFNα2 (Novusbio; NPB2-34971) for 4h to activate the *ISG15* promoter and thereby induce transcription of the monocistronic Renilla-hPEST-IRES-Puro transcript, which was followed by selection with puromycin (1 µg/mL, Thermo Fisher Scientific; A1113803) for 2 days. Cells were then subjected to limiting dilution in order to generate clonal cell populations.

Luciferase assays. The 293T cell-based luciferase assay for detection of IFN-Is has been described previously [1,3]. For detection of IFN activity by A549-IFN-reporter (AIR) cells, 30,000 cells per well were seeded in a 96-well white-bottomed tissue culture plate and stimulated 24h later with IFNα2 (Novusbio; NPB2-34971, produced in yeast cells), IFNβ (pbl assay science; 11420-1, produced in *E. coli*), IFNω (Novusbio; NBP2-35893, produced in *E. coli*), IFNγ (Novusbio; NBP2-34992, produced in *E. coli*), IFNλ1 (Novusbio; NBP2-34996, produced in *E. coli*), IFNλ2 (R&D systems; 8417-IL, produced in 293T cells), or IFNλ3 (R&D systems; 5259-IL, produced in CHO cells) at the indicated concentrations. Additionally, live-cell Renilla luciferase substrate (EnduRen, Promega; E6481) was added at a 1:10,000 final dilution. At the indicated timepoints, luciferase signal was measured using a PerkinElmer EnVision plate reader (EV2104). Data were normalized to non-stimulated control cells, and expressed as relative Renilla activity in arbitrary units.

Production of IFN subtypes in 293T cells. 150,000 293T cells seeded in 24-well tissue culture plates were transfected with 0.5 µg of each plasmid encoding the respective HiBiT-tagged IFN subtype (or GFP control) using FuGENE HD transfection reagent (Promega, E2311) according to the manufacturer's instructions. Supernatants were harvested 24h post-transfection and centrifuged at 1,000 *g* for 5 minutes to pellet cell debris. HiBiT-tagged IFNs in soluble fractions were then quantified using the Nano-Glo HiBiT Lytic Reagent Detection system (Promega) according to the manufacturer's instructions. The reconstituted luciferase activity levels were determined using a PerkinElmer EnVision plate reader (EV2104). HiBiT-normalized amounts of soluble fractions were diluted in OptiMEM containing EnduRen (1:10,000 final dilution) and used to stimulate AIR cells as described above.

IFN neutralization assays. For detection of neutralizing anti-IFN antibodies, 1:100 diluted human plasmas, a 1:100 diluted neutralizing anti-IFNγ antibody (Invitrogen, M700A), a dilution series of the indicated neutralizing anti-IFN antibodies (IFNβ, PBL assay science, 31410-1; IFNλ1: R&D systems, MAB15981; IFNλ2/3: R&D systems, MAB1587), or mock, were incubated with the appropriate IFN at the indicated concentration for 1h at room temperature with constant shaking at 600 rpm prior to addition to the appropriate reporter cell system (293T or AIR). For AIR cells, the medium was supplemented with EnduRen at a final dilution of 1:10,000. Cells were incubated for a further 24h at 37°C with 5% CO_2 prior to determination of luciferase activities as described above.

References

1. Fernbach, S.; Mair, N.K.; Abela, I.A.; Groen, K.; Kuratli, R.; Lork, M.; Thorball, C.W.; Bernasconi, E.; Filippidis, P.; Leuzinger, K.; et al. Loss of Tolerance Precedes Triggering and Lifelong Persistence of Pathogenic Type I Interferon Autoantibodies. *J Exp Med* **2024**, *221*, doi:10.1084/jem.20240365.

2. Scherrer, A.U.; Traytel, A.; Braun, D.L.; Calmy, A.; Battegay, M.; Cavassini, M.; Furrer, H.; Schmid, P.; Bernasconi, E.; Stoeckle, M.; et al. Cohort Profile Update: The Swiss HIV Cohort Study (SHCS). *Int J Epidemiol* **2022**, *51*, 33-34J, doi:10.1093/ije/dyab141.

3. Busnadiego, I.; Abela, I.A.; Frey, P.M.; Hofmaenner, D.A.; Scheier, T.C.; Schuepbach, R.A.; Buehler, P.K.; Brugger, S.D.; Hale, B.G. Critically Ill COVID-19 Patients with Neutralizing Autoantibodies against Type I Interferons Have Increased Risk of Herpesvirus Disease. *PLoS Biol* **2022**, *20*, doi:10.1371/journal.pbio.3001709.

4. Abela, I.A.; Pasin, C.; Schwarzmüller, M.; Epp, S.; Sickmann, M.E.; Schanz, M.M.; Rusert, P.; Weber, J.; Schmutz, S.; Audigé, A.; et al. Multifactorial Seroprofiling Dissects the Contribution of Pre-Existing Human Coronaviruses Responses to SARS-CoV-2 Immunity. *Nat Commun* **2021**, *12*, doi:10.1038/s41467-021-27040-x.

5. Vasou, A.; Nightingale, K.; Cetkovská, V.; Scheler, J.; Bamford, C.G.G.; Andrejeva, J.; Schwarz-Linek, U.; Randall, R.E.; McLauchlan, J.; Weekes, M.P.; et al. A Co-Opted ISG15-USP18 Binding Mechanism Normally Reserved for DeISGylation Controls Type I IFN Signalling. *BioRxiv* **2024**, doi: https://doi.org/10.1101/2021.06.01.446527.

6. Kearse, M.G.; Goldman, D.H.; Choi, J.; Nwaezeapu, C.; Liang, D.; Green, K.M.; Goldstrohm, A.C.; Todd, P.K.; Green, R.; Wilusz, J.E. Ribosome Queuing Enables Non-AUG Translation to Be Resistant to Multiple Protein Synthesis Inhibitors. *Genes Dev* **2019**, *33*, 871–885, doi:10.1101/gad.324715.119.
